# Genetic diversity and demographic history of the Old World Bollworm, *Helicoverpa armigera* (Hubner) (Lepidoptera: Noctuidae), in Ethiopia inferred from mitochondrial gene sequences

**DOI:** 10.1002/ece3.8907

**Published:** 2022-05-13

**Authors:** Tarekegn Fite, Tadele Tefera, Muluken Goftishu, Tebekew Damte

**Affiliations:** ^1^ International Centre of Insect Physiology and Ecology (ICIPE) Addis Ababa Ethiopia; ^2^ School of Plant Sciences College of Agriculture and Environmental Sciences Haramaya University Dire Dhawa Ethiopia; ^3^ 128161 Debre Zeit Agricultural Research Center Pulses, Oil and Fibre Crops Research Team Ethiopian Institute of Agricultural Research Debre Zeit Oromiya Ethiopia

**Keywords:** haplotype, migration, Old World bollworm, population expansion, population genetic structure

## Abstract

The Old World bollworm, *Helicoverpa armigera* (Hubner) (Lepidoptera: Noctuidae), is a globally distributed agricultural and horticultural insect pest. Despite the economic importance of this insect in Ethiopia, its genetic diversity and demographic history are poorly understood. We examined the nucleotide variation of the mitochondrial cytochrome c oxidase subunit I (COI) gene fragment of 74 *H*. *armigera* individuals from six collection sites in Ethiopia. We recorded 15 COI haplotypes in *H*. *armigera*, ten globally shared and five exclusive to Ethiopia (HaET15, HaET14, HaET10, HaET7, and HaET4). Haplotype HaET1 was the most widely geographically distributed and frequent (71.62%). Analysis of molecular variance (AMOVA) revealed a high and significant level of variation within *H*. *armigera* populations (*θ_ST_* = −0.0135). Negative values of the neutrality test and nonsignificant index of mismatch distribution supported the demographic expansion of H. armigera populations in Ethiopia; furthermore, this was also supported by the nonsignificant values of the sum of squared deviations (SSD) and raggedness index (r). The high genetic variation and population expansion of *H*. *armigera* have immense implications for devising locally adapted management strategies in area‐wide integrated pest management IPM programs. However, a comprehensive study of *H*. *armigera* genetic diversity and population structure using various molecular markers is needed for future confirmation.

## Graphical abstract

The TCS of *H. armigera* haplotypes based on COI gene region showing novel Ethiopian haplotypes (a) and its relation with other countries haplotypes (b)

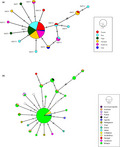

## 1 INTRODUCTION

The Old World bollworm, *Helicoverpa armigera* (Hubner) (Lepidoptera: Noctuidae), is a globally destructive insect pest (Fitt, 1989) that damages economically important crops (cotton, maize, pigeon pea, chickpea, tomato, beans, peas, sorghum, sunflower, niger seeds, etc.) (Sharma, 2005; Tebkew et al., 2002) across different ecological zones. *Helicoverpa armigera* has been reported as a key native insect pest in Ethiopia (Fite et al., 2018; Tebkew et al., 2002) and surrounding countries, such as Kenya (Kimurto et al., 2004), Tanzania (Maerere et al., 2010), and Sudan (Mansour & Mohmoud, 2014). The species has high reproductive and fecundity rates (Naseri et al., 2009; Razmjou et al., 2014) and is capable of extensive long‐distance migration (Fitt, 1989) of up to 2000 km (Behere et al., 2013; Jones et al., 2015) in a lifetime and up to 40 km in a single night (Jones et al., 2015). Up to two population peaks of *H*. *armigera* were reported in Ethiopia, beginning from June until the month of March, indicating that their population dynamics were dependent on various weather condition parameters (Fite et al., 2020). Furthermore, individuals of this species can tolerate a wide range of temperatures and drought by entering facultative diapause. Since 2013, the New World has been invaded by *H*. *armigera* (Czepak et al., 2013; Tay et al., 2013), which have Eurasian and African origins (Tay et al., 2017) and are typically confined to Africa, Europe, Asia, and Australasia (Sharma, 2005). Such high mobility with explosive population growth, tolerance to varying temperatures and drought, and a polyphagous nature have probably been important factors contributing to the establishment of this species throughout most of the world. Wide geographical distributions, the availability of many alternative host plants (Brandvain et al., 2014; Peter & Slatkin, 2013) and climate change (Bonin et al., 2007; Willi et al., 2006) all contribute to the genetic variation in organisms.

Hypothetically, high within‐population genetic variation and migration rates can provide the opportunity for new phenotypes or behaviors to emerge in pest populations (Zhou et al., 2000). Additionally, ecological parameters (Peter Linder et al., 2013), the landscape, adaptations to climate change, and resistance to environmental change (Bonin et al., 2007; Willi et al., 2006) can also impose selection pressure on crop pests. Genetic studies provide useful information regarding the potential for large‐scale insect pest control, particularly in species with extensive host ranges and wide geographical distributions (Alphey & Bonsall, 2018; Barman et al., 2012). Understanding the phylogenetic relationships among insect pest populations is critically important for informing effective and sustainable *H*. *armigera* management (Behere et al., 2007, 2013; Tay et al., 2013). However, knowledge of the population genetic diversity and demographic history of *H*. *armigera* in Eastern Africa remains poor and geographically restricted.

Due to their relatively rapid evolutionary rates and haploid mode of maternal inheritance, with little or no recombination, mitochondrial DNA sequences can be used to infer recent female‐specific evolutionary histories (Avise et al., 1987). Studies make use of the mitochondrial cytochrome c oxidase subunit I (COI) gene to distinguish natural populations of lepidopterans that have adapted to different host plants (Ong’amo et al., 2008). COI genes have been effective in studies on phylogenetic relationships, genetic variables, demographic history and phylogeography in various insects (Ajao et al., 2021; Cao et al., 2019; Xu et al., 2019), for instance, *Sesamia nonagrioides* (Lepidoptera: Noctuidae) (Goftishu et al., 2019), *Carposina sasakii* (Lepidoptera: Carposinidae) (Wang et al., 2017), and *H*. *armigera* (Tay et al., 2017). We examined the genetic diversity and demographic history of the mitochondrial COI gene fragment of 74 individuals of *H*. *armigera* from six collection sites in Ethiopia.

## 2 MATERIALS AND METHODS

### 2.1 Sampling

**FIGURE 1:**
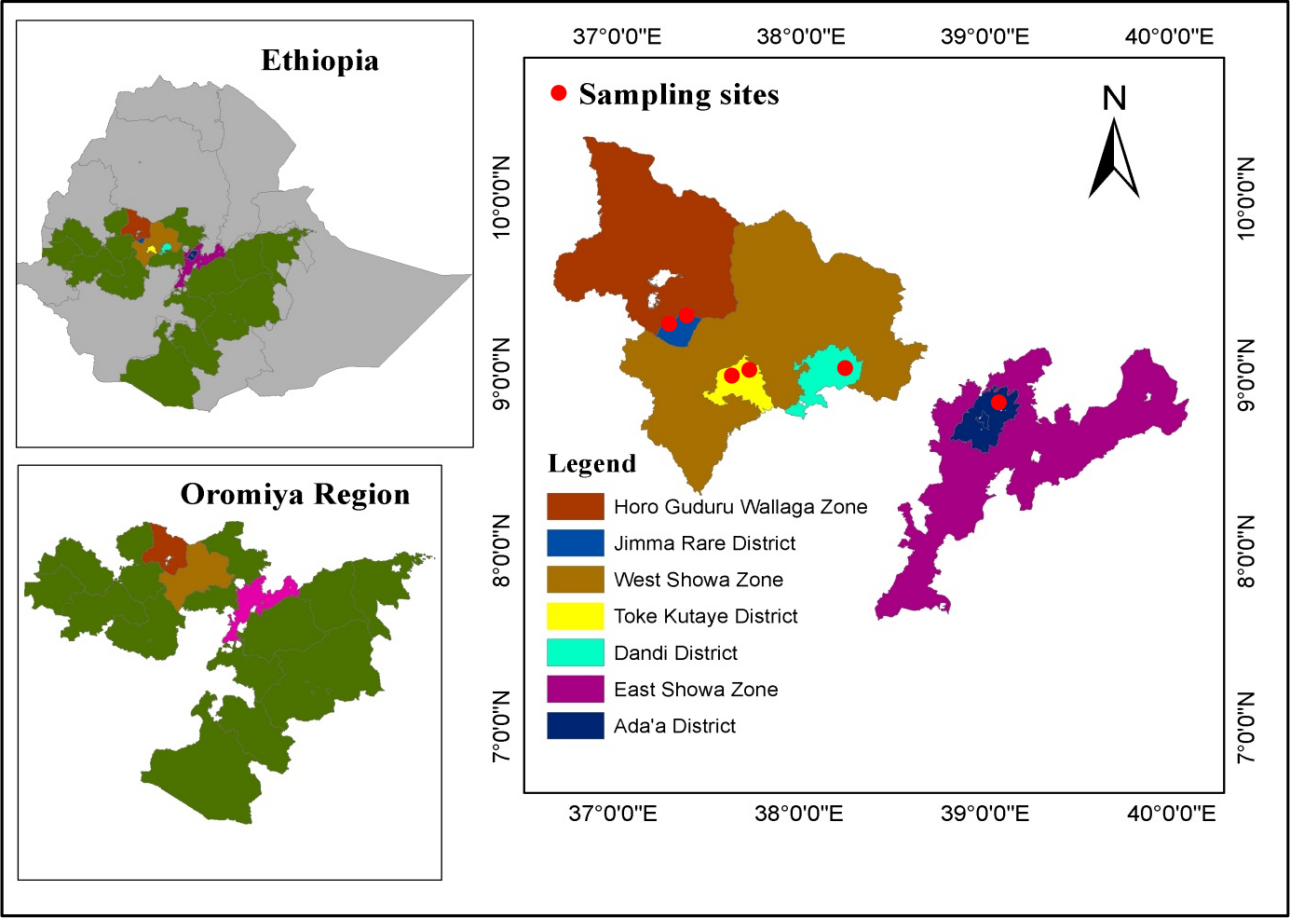
Geographical sites (districts/sampling sites) of *H*. *armigera* sampling in Ethiopia. The map was drawn using ArcGIS 10.3.1 software

Sampling was conducted in the Oromiya Regional State of central Ethiopia during the 2018 main cropping season (Figure 1). The sampling sites consisted of four districts: the Jima Rare, Toke Kutaye, and Dandi Districts located west of Addis Ababa and the Ada'a District located east of Addis Ababa. The districts are located in mountainous rivers and lake areas dominated by agricultural cultivated land. Ada'a District is located in the Great Rift Valley. The geographical distances between the sampling areas ranged between 2 and 297 km.

Sampling was carried out at the flowering stage of the respective host plants. *H*. *armigera* larvae (3rd–4th instar stages) were collected from six unsprayed host plants, chickpea (*Cicer arietinum* L.: Fabaceae) (*n* = 16), tomato (*Lycopersicon esculentum* Nill: Solanaceae) (*n* = 14), chili (*Capsicum annum* L.: Solanaceae) (*n* = 2 due to low sequence quality), niger (*Guizotia abyssinica* L.: Asteraceae) (*n* = 12), sunflower (*Helianthus annuus* L.: Asteraceae) (*n* = 13), and peas (*Pisum sativum* L.: Fabaceae) (*n* = 17), across the four districts during the 2018 main cropping season in the Oromiya Regional State of central Ethiopia (Table 1). A total of 74 *H*. *armigera* larvae were used for the analysis of genetic diversity and demographic history (Table 1).

**TABLE 1:** Information on the sample collection description used for the study and available NCBI GenBank accessions of *H*. *armigera* from Ethiopia (*n* = sample size)

Population Code	*n*	Districts	Latitude	Longitude	Elevation (m)	Host Plants	GenBank accessions
ChAD	16	Ada'a	08°41.570′	039°03.545′	1865	Chickpea	MW520816‐MW520825
TTK	14	Toke Kutaye	08°59.316′	037°47.398′	1956	Tomato	MW520752‐MW520765
CTK	2	Toke Kutaye	08°56.316′	037°42.398′	1908	Chili	MW520808‐MW520809
SDD	13	Dandi	09°01.303′	038°07.094′	2285	Sunflower	MW520766‐MW520778
NJR	12	Jima Rare	09°18.520′	037°20.87′	2309	Niger	MW520796‐MW520807
PJR	17	Jima Rare	09°18.780′	037°20.39′	2230	Peas	MW520779‐MW520795
Total	74						

Abbreviations: ChAD, populations collected from chickpea; CTK, populations collected from chili; NJR, populations collected from niger; PJR, populations collected from pea; SDD, populations collected from sunflower; TTK, populations collected from tomato.

The collected larvae were kept in plastic vials for 24 h for starvation. Then, they were preserved in absolute ethanol and labeled with their host plants, site, date of collection, and GPS coordinates and stored at −20°C until required for DNA extraction.

### 2.2 DNA extraction

Genomic DNA was extracted using the established protocols described by Behere et al. (2013) with modifications. Briefly, the absolute ethanol‐preserved specimens were washed with sterilized distilled water and kept on a paper towel for 10 min at room temperature to allow the ethanol to evaporate and the insects to dry. Insect material consisting of the head and/or posterior end or whole larval instars estimated to weigh 100 mg was cut off with sterilized surgical blades. The cleaned larvae were ground in liquid nitrogen and genomic DNA was extracted using a Genomic II DNA Extraction Kit (BIOLINE) following the manufacturer's instructions. Genomic DNA was visualized on a 1% agarose gel for detection and an Eppendorf BioSpectrometer (Germany) to check the quality and quantity of the extraction protocols before being used for polymerase chain reaction (PCR).

### 2.3 PCR amplification and sequencing

We amplified a 511‐bp fragment of the COI gene by PCR in 10‐μl reaction volumes containing 6 μl of nucleus‐free water, 2 μl of 5× HOT FIREPol^®^ Blend Master Mix (Solis BioDyne), 0.5 μl of each 10 µM primer (COI‐F02: 5′‐CTCAAATTAATTACTCCCCATC‐3′; COI‐R02: 5′‐GGAGGTAAGTTTTGGTATCATT‐3′) (Behere et al., 2013), and 1 μl of template DNA. PCR was run under the following conditions: initial denaturing at 95°C for 15 min, followed by 40 cycles of denaturation at 95°C for 30 s, annealing at 53°C for 30 s, and extension at 72°C for 1 min, followed by a final extension at 72°C for 10 min. The amplified PCR products were sequenced by Macrogen Inc. after purification by an Exo 1‐rSAP combination (Biolabs) according to the manufacturer's protocol.

### 2.4 Sequence analyses

DNA sequences were aligned in MAFFT v 7.450 (Katoh & Daron, 2013) using Geneious Prime, 2020.2.3 software (Biomatters, Ltd) (https://www.geneious.com), and the alignments were verified visually. A TCS network (Clement et al., 2002) was constructed with the program POPART (Leigh & Bryant, 2015) to investigate the possible relationships among haplotypes. Related sequences were identified by querying the GenBank nr database using the Basic Local Alignment Search Tools implemented in Geneious Prime software (version indicated above). The resulting 402‐bp fragment of the COI sequences from each population sampled in this study was aligned with 26 *H*. *armigera* COI sequences retrieved from the National Center for Biotechnology Information (NCBI) GenBank (www.ncbi.nlm.nih.gov/genbank/) repository by blasting the gene sequences for TCS network construction. These sequences were obtained from 14 countries: one each from the Dominican Republic (accession: MK645066 – Luke et al., 2019), Uganda (MK645209 – Luke et al., 2019), Spain (KX494897 – Tay et al., 2017), France (KX494893 – Tay et al., 2017), Brazil (KX494899 – Tay et al., 2017), Senegal (KX494879 – Tay et al., 2017), and Cameroon (KX494886 – Tay et al., 2017); two each from Ghana (KX494882 and KX494881 – Tay et al., 2017), Kenya (MK645094 and MK645092 – Luke et al., 2019), Zimbabwe (MK645219 and MK645220 – Luke et al., 2019), and South Africa (MK645215 and MK645214 – Luke et al., 2019); three each from Madagascar (KX494884, KX494885, and KX494883 – Tay et al., 2017) and Chad (KX494888, KX494887, and KX494894 – Tay et al., 2017); and five from Australia (EF116235, EF116246, EF116227, EF116240, and EF116229 – Behere et al., 2007).

All the newly sequenced samples were deposited in the GenBank database of the NCBI.

The number of haplotypes (*H*), haplotype diversity (*H_d_*), and nucleotide diversity (*pi*) were used to estimate genetic variability between haplotypes using DnaSP v. 5.10 program (Librado & Rozas, 2009). Arlequin v. 3.5.2.2 (Excoffier & Lischer, 2010) software was used to investigate the demographic history of the populations: Tajima's D Tajima (Tajima, 1989a) and Fu's *Fs* (Fu, 1997) were estimated among populations. The spatial expansion hypothesis of Harpending's raggedness index (r) (Harpending et al., 1993) and the sum of squared deviations (SSD) were also tested using a parametric bootstrap approach with 1000 replicates using Arlequin (version indicated above). Similar software was used to test the hierarchical genetic structure of the populations based on analysis of molecular variance (amova).

## 3 RESULTS

### 3.1 Genetic variation and diversity

We generated 402‐bp mtDNA COI gene sequences of 74 *H*. *armigera* specimens obtained from the six populations across the sampling sites, all of which shared 99%–100% homology with the reference *H*. *armigera* sequences found in GenBank (https://www.ncbi.nlm.nih.gov/genbank/) (Table 1). All the sequenced samples were deposited in the GenBank database of the NCBI under accession numbers MW520752‐MW520765 (TTK), MW520766‐MW520778 (SDD), MW520779‐MW520795 (PJR), MW520796‐MW520807 (NJR), MW520808‐MW520809 (CTK), and MW520810‐MW520825 (ChAD) (Table 1).

For each of the population characteristics, polymorphisms were found in all six populations, which ranged from two for NJR to nine for PJR. The overall total polymorphic site number was 14. However, on a population basis, PJR had the most (9) polymorphic sites, followed by the ChAD and TTK populations (Table 2). The sequenced partial portion of the COI mtDNA gene region revealed the presence of a total of 15 haplotypes from all six populations of H. armigera in Ethiopia (Table 2). The number of haplotypes per population ranged from two for CTK to seven for PJR. Among the six populations, the CTK population had the highest (1.000) haplotype diversity (*H_d_*), which could be attributed to its low sample size, followed by PJR (Table 2). The *H_d_* ranged from 0.318 to 1.000 with an average of 0.486, while the nucleotide diversity (*pi*) ranged from 0.00083 to 0.00329 with an average of 0.00242 (Table 2).

**TABLE 2:** Parameters of the genetic diversity test based on of *H*. *armigera* COI mtDNA sequence data from six populations in Ethiopia

Population code	*n*	Number of polymorphic sites (*s*)	Number of haplotypes (*H*)	Haplotype diversity (*H_d_*)	Nucleotide diversity (*pi*)
ChAD	16	7	5	0.533	0.00325
TTK	14	5	4	0.396	0.00208
CTK	2	1	2	1.00	0.00249
SDD	13	4	4	0.526	0.00217
NJR	12	2	3	0.318	0.00083
PJR	17	9	7	0.596	0.00329
Total	74	14	15	0.486	0.00242

Abbreviation: *n*, sample size.

### 3.2 Haplotype distribution and network

**FIGURE 2:**
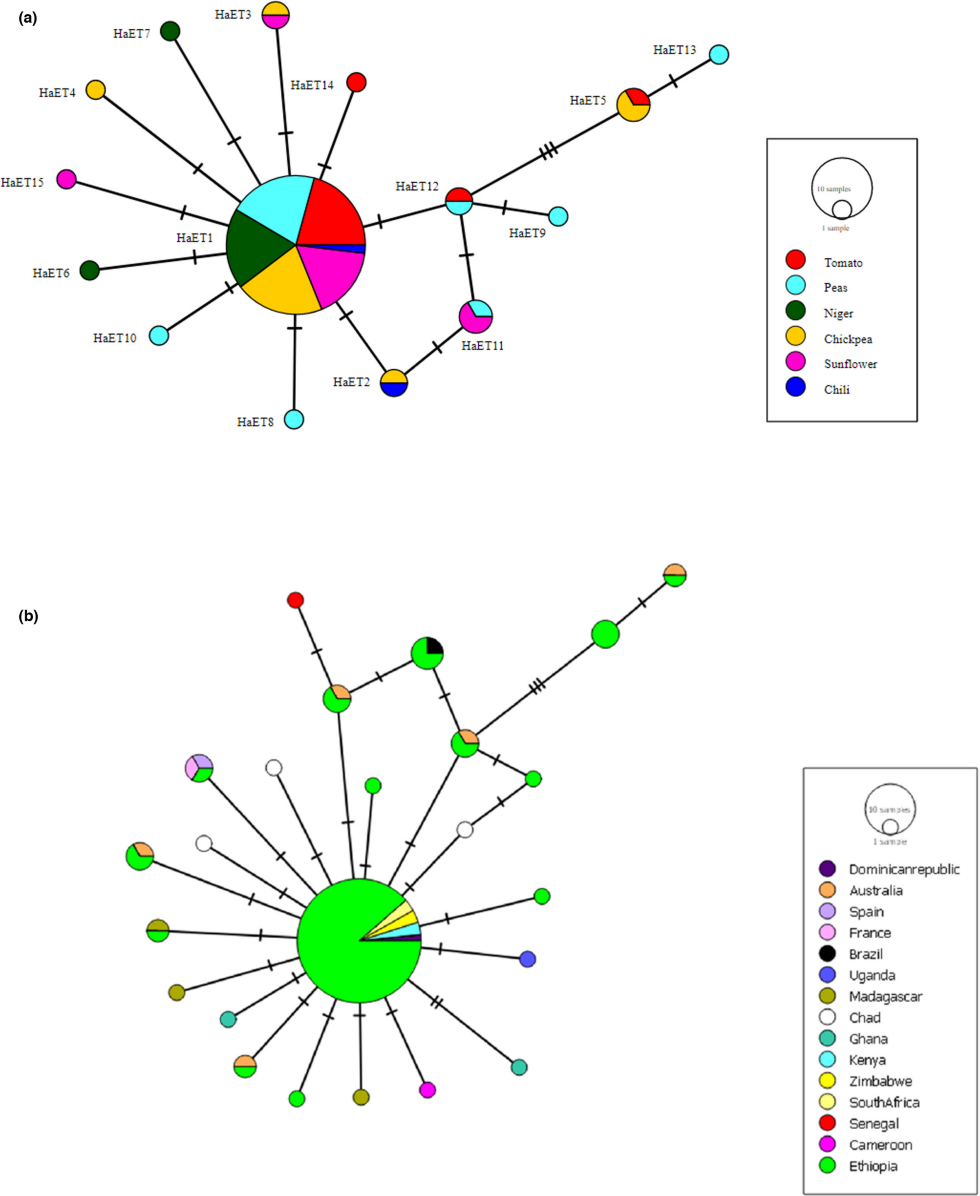
TCS network of *H*. *armigera* haplotypes based on the COI gene region showing novel Ethiopian haplotypes (a) and their relations with haplotypes from other countries (b). Each circle represents a haplotype, and the circle size is proportional to haplotype frequency. Colors indicate the proportion of individual samples in different populations for the host plants (a) and countries (b). Tick marks between haplotypes represent single nucleotide polymorphisms

A total of 15 *H*. *armigera* haplotypes were identified from the alignment of all 74 COI gene sequences from the six populations, of which five haplotypes were HaET4 from the ChAD population, HaET7 from the NJR population, HaET10 from the PJR population, HaET14 from the SDD population and HaET15 from the TTK populations, which were represented by a single individual (Table 3). These haplotypes (HaET15: MW520752, HaET14: MW520768, HaET10: MW520781, HaET7: MW520796, and HaET4: MW520812) identified in Ethiopia were unique to their populations. The most common haplotype (HaET1) was found in 53 individuals, accounting for 71.62% of the total individuals, was evenly distributed in the six populations of *H*. *armigera* in Ethiopia and occupied the central region of the haplotype network (Table 3, Figure 2a). The Ethiopian haplotypes differed by 1–7 mutational steps from the ancestral haplotype, showing a star‐like expansion pattern in *H*. *armigera* (Figure 2a). The haplotype network analysis of COI gene sequences of *H*. *armigera* from Ethiopia revealed similarities with multiple individuals from other countries (Figure 2b). Most of the Ethiopian individuals occupied the central position (common central haplotype) with individuals from four countries, South Africa, Zimbabwe, Kenya, and the Dominican Republic, in the haplotype network, suggesting a large geographical distribution and a higher frequency of Ethiopian haplotypes in the dataset. Furthermore, many of the haplotypes migrated from Ethiopia into other countries (Ghana, Madagascar, Chad, Cameroon, and Uganda), continents (Europe (Spain and France)), (South America (Brazil)), and the Caribbean region (Dominican Republic)). Generally, most of the haplotypes differed by 1–5 mutational steps from the central ancestor haplotype (Figure 2b).

**TABLE 3:** COI haplotype distribution in each population of *H*. *armigera* in Ethiopia

Haplotypes	ChAD	TTK	NJR	CTK	PJR	SDD	Total number
HaET1 [**Global**]	11	11	10	1	11	9	53
HaET2 [**Harm10**]	1			1			2
HaET3 [**Harm21**]	1					1	2
HaET4^a^	1						1
HaET5 [**Harm03**]	2	1					3
HaET6 [**Harm15**]			1				1
HaET7^a^			1				1
HaET8 [**Hap48**]					1		1
HaET9 [**Hap39**]					1		1
HaET10^a^					1		1
HaET11 [**Hap54**]					1	2	3
HaET12 [**Harm02**]		1			1		2
HaET13 [**Harm04**]					1		1
HaET14^a^						1	1
HaET15^a^		1					1
Total	16	14	12	2	17	13	74

Note

Black bold text indicates *H*. *armigera* haplotypes matching Ethiopian haplotypes.

^a^ Unique haplotypes, named with new Ethiopian names and deposited in the GenBank database of the National Center for Biotechnology Information (NCBI).

### 3.3 Population structure and demographic history

amova was performed to determine how the genetic variability was distributed among and within the populations (Table 4). amova did not suggest hierarchical genetic structure among the populations (Table 4). However, a high and significant percentage (101.35%) of the total variation occurred within populations (Table 4).

**TABLE 4:** Analysis of molecular variance (amova) results for six populations of *H*. *armigera* based on variation of mtCOI gene sequences

Source of variation	df	Sum of squares	Variance components	Variation (%)	Fixation indices	*p*‐value
Among populations	5	2.066	−0.007va	−1.35	*F_SC_* = 0.000	0.615 ± 0.017
Within populations	63	33.393	0.491vb	101.35	θ*_ST_* = −0.0135	0.000 ± 0.000
Total	73	35.459	0.484	100		

Note

Fixation indices for among populations (*F_SC_*) and within populations (θ*_ST_*).

Abbreviation: df, degrees of freedom.

Based on the overall COI neutrality tests, the results were nonsignificantly (*p *> .05) negative, with values of −1.220 (Tajima's D value), indicating deviation from evolutionary neutrality, and −1.102 (Fu's *F_s_* values), indicating an excess of rare haplotypes in the population compared to what is expected under a neutral model of evolution (Table 5).

**TABLE 5:** Neutrality test and mismatch distribution analysis based on six populations of *H*. *armigera* COI mtDNA sequence data in Ethiopia

Population code	Tajima's D test (*p*‐value)	Fu's *F_s_* test (*p*‐value)	SSD (*p*‐value)	*r* (*p*‐value)
ChAD	−1.338 (.100)	−0.752 (.229)	.032 (.280)	.153 (.730)
TTK	−1.623 (.045)	−0.812 (.147)	.015 (.350)	.209 (.660)
CTK^a^	.000 (1.00)	.000 (.245)		
SDD	−1.09932 (.148)	−0.810 (.140)	.006 (.670)	.099 (.830)
NJR	−1.451 (.085)	−1.325 (.004)	.003 (.460)	.226 (.590)
PJR	−1.812 (.018)	−2.915 (.014)	.010 (.600)	.067 (.890)
Total	−1.220 (.232)	−1.102 (.129)	.011 (.393)	.126 (.616)

Abbreviations: SSD, sum of squares deviation; r, raggedness index.

^a^ Values not estimated due to low sample size.

The mismatch distribution was unimodal for the Ethiopian populations of *H*. *armigera*, indicating that rare alleles present at high frequencies possibly contributed to population expansion and/or selection. Furthermore, this result was supported by the nonsignificant (*p *> .05) values of SSD and raggedness index for all (except the TKC population) of the populations, indicating the presence of nonequilibrium and a population expansion event, and the data have a relatively good fit to a model of population expansion in *H*. *armigera* (Table 5).

## 4 DISCUSSION

### 4.1 Genetic variation and diversity

Correct identification and a thorough understanding of the genetic variation of an insect pest are essential in the development and improvement of monitoring and pest management strategies (Assefa et al., 2017). Four species of Helicoverpa occur in Africa: The oriental tobacco budworm, *Helicoverpa assulta* (Lepidoptera: Noctuidae), which occurs in many areas of Africa, including Kenya, Cameroon, South Africa, Tanzania, and Uganda; the *H*. *fletcheri* (Hardwick) (Lepidoptera: Noctuidae), which was reported from Africa, including Sudan (Hackett & Gatehouse, 1982); and *H*. *peltigera* (Schiffermuller) (Lepidoptera: Noctuidae), which is also present in most of Africa, such as Eritrea, Egypt, Sudan, Algeria, and Chad (Ahmed & Elamin, 1996), and the most economically important and widely distributed, *H*. *armigera*. Hence, the present investigation provides an efficient way to identify Helicoverpa species in the country. All sampled individuals from the six populations were confirmed to be *H*. *armigera*, representing the first molecular characterization of *H*. *armigera* in Ethiopia using the COI mtDNA gene region. DNA barcoding, such as using COI mtDNA, is an applicable and efficient method for the separation and confirmation of insect species (Foottit et al., 2008; Jung et al., 2010), including *H*. *armigera* (Leite et al., 2014).

Our data indicated lower haplotype diversity and higher nucleotide diversity when compared to several previous reports, reflecting the presence of few segregating sites across different haplotypes of the populations. In populations of *H*. *armigera*, haplotype diversity (*H_d_*) and nucleotide diversity (*pi*) values of 0.821 and 0.0028, respectively, were detected by Leite et al. (2014) in Brazil, and values of *H_d_* = 0.765 and *pi* = 0.0021 were reported by Wang et al. (2018) in China; these values are higher than the haplotype diversity value we obtained in Ethiopia (*H_d_* = 0.486). Recently, Arnemann et al. (2019) reported varying haplotype and nucleotide diversities on different continents (Asia: *H_d_* = 0.912 and *pi* = 0.00360, Europe: *H_d_* = 0.738 and *pi* = 0.00238, Australia: *H_d_* = 0.882 and *pi* = 0.00403, South America: *H_d_* = 0.769 and *pi* = 0.00244). The low haplotype diversity and nucleotide diversity observed among *H*. *armigera* populations in the present study can be explained by the dispersal capacity (López & Marisa, 2017) and demographic expansion of *H*. *armigera* (Sosa‐Gómez et al., 2016). Similarly, Behere et al. (2007) also reported low nucleotide diversity (*pi* = 0.0017–0.0038) between countries when using similar molecular markers, which they stated could be due to the high mobility in *H*. *armigera*. Additionally, the low haplotype diversity in our findings also reflects the small number of *H*. *armigera* specimens and populations used.

### 4.2 Haplotype distribution and network

We detected a total of 15 haplotypes in Ethiopian *H*. *armigera* populations using COI gene sequences. The majority of the haplotypes were randomly distributed throughout the six populations. The presence of unique haplotypes across the populations may be due to the small sample size used in this study. Leite et al. (2014) identified 31 haplotypes from Brazilian populations using the COI gene sequence of *H*. *armigera*, while the global COI gene sequence was higher (33 haplotypes) (Behere et al., 2007). The network clearly showed a star‐like expansion pattern for Ethiopian *H*. *armigera* haplotypes. This star‐like haplotype networks in Ethiopian haplotypes, which were ancestral haplotypes for most of the compared haplotypes from various countries, indicate invasion events between those countries. It was evident that the Old World was the source of infestation of *H*. *armigera* to the New World, as several maternal lineages are prevalent throughout the Old World (Tay et al., 2013). Similarly, signs of demographic expansion were found for *H*. *armigera* within South America (Goncalves et al., 2019) and Brazil (Leite et al., 2014). In particular, Goncalves et al. (2019) reported Europe as the origin of South American specimens of *H*. *armigera* following a northward movement through the Caribbean based on the COI gene fragment. Several studies have found unusually high levels of genetic variation both within native populations of *H*. *armigera* and within *H*. *armigera* populations that have been introduced to South America (Leite et al., 2014; Tay et al., 2017), likely contributing to its very high adaptive and invasive capacities (Anderson et al., 2016).

### 4.3 Population structure and demographic history

The weak population structure observed among the populations of *H*. *armigera* suggests that the variation was distributed randomly between populations due to the high gene flow resulting from migrations of the insect. Our results showed a lack of differentiation among the six studied populations of *H*. *armigera*, which was in line with reports from China, India, and Australia (Kraus et al., 2011; Scott et al., 2005; Weeks et al., 2010) and indicated that gene flow has been high enough to prevent trade‐offs in fitness between *H*. *armigera* populations attacking diverse host plants from creating isolation. Based on several molecular markers, including allozymes, microsatellites and mtDNA, unstructured genetic networks of *H*. *armigera* distributed in other regions have been reported (Endersby et al., 2007; Nibouche et al., 1998). Therefore, the genetic variation observed in this study is not solely associated with differences in sampling sites. The biological characteristics allowed extensive movement of *H*. *armigera* with similar genetic make‐ups/genetic homogeneity and allowed the insects to breed across the geographical populations we examined in Ethiopia. Compared to host‐monophagous insects, polyphagous herbivorous insects such as *H*. *armigera* exhibit extensive plasticity in feeding depending on the host plant (Wang et al., 2017).

Demographic analysis using neutrality tests and a mismatch distribution analysis with COI mtDNA markers revealed expansion of *H*. *armigera* within the Ethiopian territory; a similar result was reported in Brazil by Leite et al. (2014) on the basis of similar molecular markers. When a genetic structure has been influenced by rapid range expansion, Tajima's D is expected to be negative, indicating an excess of rare nucleotide variants compared to the expected value under a neutral model of evolution Tajima (1989b). Similarly, Fu's *F_s_* test, which is based on the distribution of haplotypes, also showed negative values for *H*. *armigera* populations in Ethiopia, confirming an excess of rare haplotypes over the number that would be expected under neutrality (Fu, 1997). The nonsignificant values of SSD and r also support this interpretation. The observed nonsignificant values in goodness of fit distribution suggest that population expansion occurred recently (Rogers & Harpending, 1992) in populations of *H*. *armigera* in Ethiopia, which is also supported by the high within‐population genetic variation and unique haplotypes observed in the present finding. A similar situation was previously found in insect species, with a high migration rate and from a small effective population size (Kraus et al., 2011), including insect pests such as *Plutella xylostella*, as inferred from COI mtDNA (Wei et al., 2013). The present population expansion of *H*. *armigera* in Ethiopia could also be explained by the diverse agro‐ecological variations, weather‐based population dynamics, availability of host plants year‐round, and the expansion of certain host plants, such as tomato chickpea production, which has provided more food options and ecological environments for *H*. *armigera*. The developmental rate, survivorship, reproduction, and life table parameters of *H*. *armigera* could be affected by the different nutritive values of various host plants (Razmjou et al., 2014). The extensive commercial cultivation of tomato and chickpea in recent years may have also contributed to the expansion of the *H*. *armigera* distributed in Ethiopia.

### 4.4 Implications in pest management

The results of this study indicate a relatively low genetic diversity and demographic expansion of *H*. *armigera* within Ethiopian territory. Such demographic expansion of *H*. *armigera* will likely affect the sustainable management of *H*. *armigera* in Ethiopia, due to the high dispersion capacity of *H*. *armigera*. Thus, any allele fixed by genetic drift or selection in a region or host can spread to other Ethiopian areas and other countries. This scenario requires serious attention and may inform locally adapted management strategies in integrated pest management (IPM) programs in the region.

### 4.5 Conclusions

Understanding the genetic diversity and demographic history of *H*. *armigera* populations is critically important for designing effective and sustainable *H*. *armigera* management programs. Our results revealed a total of 15 *H*. *armigera* haplotypes for the COI gene sequences of 74 individuals from six populations with lower haplotype diversity and higher nucleotide diversity. Demographic analysis using neutrality tests and a mismatch distribution analysis with COI mtDNA markers revealed expansion of *H*. *armigera* within the Ethiopian territory. However, a comprehensive study on *H*. *armigera* genetic diversity and population structure using various molecular markers is needed for future confirmation.

## AUTHOR CONTRIBUTIONS

**Tarekegn Fite:** Conceptualization (equal); Data curation (equal); Formal analysis (equal); Investigation (equal); Methodology (equal); Software (equal); Validation (equal); Visualization (equal); Writing – original draft (equal); Writing – review & editing (equal). **Tadele Tefera:** Conceptualization (equal); Funding acquisition (lead); Project administration (equal); Resources (equal); Supervision (equal); Validation (equal); Writing – review & editing (equal). **Muluken Goftishu:** Data curation (equal); Methodology (equal); Supervision (equal); Writing – review & editing (equal). **Tebekew Damte:** Conceptualization (equal); Data curation (equal); Supervision (equal); Visualization (equal); Writing – original draft (equal); Writing – review & editing (equal).

## CONFLICT OF INTEREST

The authors declare no conflicts of interest.

## Data Availability

The sequence used in this study are deposited in the GenBank database of National Center for Biotechnology Information (NCBI) under the accession numbers (MW520752‐MW520765) (TTK), (MW520766‐MW520778) (SDD), (MW520779‐MW520795) (PJR), (MW520796‐MW520807) (NJR), (MW520808‐MW520809) (CTK), and (MW520810‐MW520825) (ChAD).
